# Attraction of cabbage stem flea beetle ( *Psylliodes chrysocephala* ) to host plant odors

**DOI:** 10.1002/ps.8697

**Published:** 2025-02-04

**Authors:** Daniel Rüde, Bernd Ulber, Samantha M. Cook, Michael Rostás

**Affiliations:** ^1^ Agricultural Entomology, Department of Crop Sciences University of Göttingen Göttingen Germany; ^2^ Protecting Crops and the Environment Rothamsted Research Harpenden UK

**Keywords:** *Psylliodes chrysocephala*, *Brassica napus*, *Sinapis alba*, *Brassica rapa*, olfactometer, isothiocyanates

## Abstract

**BACKGROUND:**

Oilseed rape (OSR, *Brassica napus*) faces substantial yield losses in Europe due to the cabbage stem flea beetle (CSFB, *Psylliodes chrysocephala*). Synthetic insecticide use is constrained by resistance and environmental concerns, necessitating innovative pest control strategies. Understanding CSFB host plant selection, particularly through volatile organic compounds (VOCs), is essential for developing sustainable and efficient methods. This study investigated the olfactory response of CSFB in their pre‐aestivation stage to plant VOCs.

**RESULTS:**

Olfactometer bioassays showed that female CSFB were attracted to VOCs from mechanically damaged OSR plants (BBCH 10 and BBCH 14), while undamaged OSR plants elicited no response. Damaged seedlings of *Sinapis alba* and *Brassica rapa* were not attractive. When testing individual isothiocyanates, again only female CSFB showed a positive response in the olfactometer bioassays, while no response was found for two green leaf volatiles.

**CONCLUSION:**

This research provides insights into the olfactory behavior of CSFB and, to our knowledge, is the first to show behavioral responses of adult CSFB towards host plant volatiles in olfactometer tests. Interestingly, only females responded to VOCs, suggesting sexual dimorphism in olfactory sensitivity during this life stage. These findings may help to lay the groundwork for further studies aimed at improving pest management strategies in OSR cultivation. © 2025 The Author(s). *Pest Management Science* published by John Wiley & Sons Ltd on behalf of Society of Chemical Industry.

## Graphical abstract

Oilseed rape (*Brassica napus*, OSR) cultivation in Europe is threatened by the cabbage stem flea beetle (*Psylliodes chrysocephala*, CSFB). Limited management options due to resistance and environmental concerns necessitate innovative control strategies. This study explores CSFB responses to volatile organic compounds (VOCs) emitted by host and non‐host plants. Olfactometer bioassays revealed significant attraction to damaged OSR, with isothiocyanates playing a key role as host plant cues. Notably, only female CSFBs responded to VOCs, suggesting sexual dimorphism in olfactory sensitivity. These findings provide novel insights into CSFB behavior and contribute to developing sustainable, integrated pest management strategies for OSR.

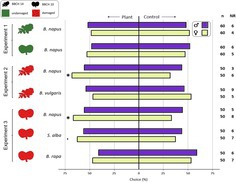

## 1 INTRODUCTION

Oilseed rape (OSR, *Brassica napus*) is the most important oil crop grown in Europe [European Commission (https://agridata.ec.europa.eu)]. The crop faces severe threats from various insect pests, with the cabbage stem flea beetle (CSFB, *Psylliodes chrysocephala* L.) being one of the most destructive.^1^, ^2^ Adult CSFB feed on very young OSR plants in autumn, while larvae mine within the petioles and stems until pupation in spring.^3^ Currently, the control of CSFB relies on synthetic insecticides, mainly pyrethroids, after the ban of neonicotinoids. Due to increasing resistance problems and restrictions on the use of insecticides, there is a pressing need for effective control strategies.^1^, ^4^

A key challenge in developing ecology‐based efficient control methods for CSFB is the limited understanding of their host plant selection behavior. Thorough knowledge of the detection and behavioral response of CSFB to volatile organic compounds (VOCs) emitted by host plants could provide valuable insights into the cues required for location and acceptance of suitable hosts.^4^ This would facilitate the development of several components for integrated pest management. A better understanding of the role of olfactory cues is required for the development of more effective monitoring and/or control strategies, such as push‐pull or the design of baited traps.^5^, ^6^ Additionally, emitted volatiles represent a possible target for breeding insect pest resistant cultivars.^4^ In the case of *Brassica napus*, the presence of resistance to insect herbivores in this species is suspected to be limited, owing to its constrained genetic diversity.^7^ Consequently, the identification of resistance donors from related species, such as *Sinapis alba*, becomes necessary.^4^, ^8^

The CSFB is considered a specialist on Brassicaceae.^9^, ^10^ The mechanisms by which CSFB locates OSR crops after aestivation remain uncertain, though several possibilities have been proposed. These include olfactory detection, the use of visual cues, and chance encounters. Additionally, CSFB may orient themselves using color contrasts.^11^ It is also conceivable that CSFB employ a combination of olfactory and visual signals to locate OSR host plants.^12^, ^13^ Previous studies have shown the involvement of isothiocyanates (ITCs) as stimuli for CSFB. ITCs are VOCs produced by enzymatic conversion of glucosinolates and are specific for Brassicaceae.^14^ Studies on the olfactory system of CSFB have revealed that certain ITCs elicit electrophysiological activity in the antennae of adult CSFB.^15^, ^16^ This indicates that these specific ITCs can be detected by the beetles and may play a role in guiding their behavior. Further field investigations demonstrated that water traps baited with a mixture of ITCs increased CSFB catches, indicating the positive response to these *Brassica*‐specific VOCs.^17^ However, to our knowledge, experiments investigating the behavioral response of CSFB to VOCs using olfactometer bioassays have not been published so far. This lack of data is a critical knowledge gap, since olfactometer experiments are essential for investigating the chemical ecology of plant–insect interactions.^18^, ^19^ Unlike field tests, olfactometer assays offer a highly controlled environment through standardization, ensuring replicability and enabling precise quantification of the effects of specific test compounds.

Finding a suitable olfactometer setup for a specific test species can be challenging, due to the many factors that need to be considered.^19^ Therefore, the first objective of this study was to establish an effective olfactometer assay under laboratory conditions to examine the behavioral response of CSFB to plant VOCs. We focused on the seedling stage of the plants, which is particularly vulnerable to feeding by adult CSFB and because little is known about the VOC emissions of brassicaceous seedlings. Three different Brassicaceae species were tested in the olfactometer, with *Brassica napus* (OSR) as the main crop plant, *S. alba* as a possible resistance donor, and *Brassica rapa* as a species presumed to be highly susceptible.^8^, ^20^, ^21^ All three species are recognized as potential hosts for both CSFB larvae and adults. However, differences in susceptibility to CSFB feeding are anticipated.^8^, ^9^ This variation in susceptibility raises the question: Does the odor emitted by different Brassicaceae species vary in attractiveness to CSFB? Understanding this could have valuable implications for breeding resistant cultivars and enhancing integrated pest management strategies. *Beta vulgaris*, which is also a widely cultivated dicotyledonous crop plant in northern Europe,^22^ was chosen as a non‐host. After identifying the VOC profiles of the test plants, the attractiveness of individual compounds was tested in olfactometer assays and in a field experiment. The field assay, using baited traps, was completed to validate the olfactometer experiments in the laboratory and to check whether baiting with individual volatiles could increase beetle catches in conventional monitoring traps.

## 2 MATERIALS AND METHODS

### 2.1 Insects

Adult CSFB were obtained by extracting larvae from field‐grown OSR plants and rearing them until they emerged as adults. OSR (cv. Bender) plants were collected between January and April 2023 in a field in Göttingen, Germany (coordinates: 51°33'56.4"N 9°56'45.8"E). To extract larvae from OSR, the plants were placed into plastic containers (37 cm × 26.5 cm × 13.2 cm, BPA free) which had a perforated bottom (60 holes of 10 mm diameter) and were sealed with perforated clingfilm as a lid. Third instar (L_3_) larvae were collected daily from the plastic box attached below the extraction container and placed in plastic containers filled with humid substrate, in which pupation took place. The substrate was composed of a 1:1:1 (by volume) mixture of sand (0.1 mm–2 mm diameter; Oppermann Kiesgewinnungs‐ und Vertriebs‐GmbH, Hannoversch Münden, Germany), potting soil (Fruhstorfer Erde Type P 25; HAWITA GmbH, Vechta, Germany), and loam (obtained from field subsoil in Pöhlde, Germany). The containers were stored at 10 °C. For the experiments, the containers were transferred to a controlled climate room [16 ± 1 °C, 60.4 ± 6% relative humidity (RH)] with day light spectrum light‐emitting diodes (LEDs, GoLeaf E2; DEL‐KO GmbH, Augsburg, Germany) to stimulate eclosion of the adults. Emerging adults were collected daily and used in experiments within 3–6 days. Until use, adult beetles were kept in net cages (24.5 cm × 24.5 cm × 24.5 cm; BugDorm‐4 M2222, Megaview Science Co., Ltd, Taichung, Taiwan) and supplied with fresh OSR leaves (cv. Arabella). Beetles were starved for 24 h prior to the experiments and their sex was determined according to Cook *et al*.^23^ In this study, only pre‐aestivated beetles were used because rearing them from field‐collected larvae ensured a high level of standardization regarding their nutritional status, motivation, and age. In contrast, post‐aestivated beetles could not be captured in sufficiently high numbers in the field and would exhibit significant variability in terms of age, prior feeding, and oviposition status.

### 2.2 Plants

The plants tested in the olfactometer assays were OSR – *Brassica napus* cv. Arabella (Limagrain GmbH, Edemissen, Germany), *S. alba* cv. Passion (Deutsche Saatveredelung AG, Lippstadt, Germany), *Brassica rapa* cv. Jupiter (Saaten‐Union, Isernhagen, Germany) and *Beta vulgaris* subsp. *vulgaris* cv. Vasco (SESVanderHave, Würzburg, Germany). Seeds were sown in plastic pots (10 cm × 12 cm) containing a 1:1 mixture of sand and potting soil as used before. Seedlings were cultivated in a controlled environment room (20.2 ± 1 °C, 59–61% RH) under high pressure sodium lamps (HS.TP400; Hortilux Schréder, Monster, The Netherlands) with a photoperiod of 16 h:8 h (light/dark) and used when their cotyledons had fully expanded (BBCH 10), which typically occurred 5–6 days after sowing. To obtain older plants, *Brassica napus* and *Brassica vulgaris* seedlings were transplanted into pots containing a 1:2 mixture of sand and potting soil. The pots were placed in a glasshouse with high pressure sodium lamps, and the plants were grown until they reached the 3–4 true leaf stage (BBCH 14). Control pots without plants were prepared using the same soil mixture and were maintained under the same conditions as the experimental plants.

### 2.3 Olfactometer assays

We tested the behavioral responses of adult CSFB to various odors, including those from undamaged OSR seedlings (BBCH 10) and plants in the four‐leaf stage (BBCH 14), damaged OSR plants at BBCH 14, damaged non‐host *Beta vulgaris* at BBCH 14, and seedlings (BBCH 10) of OSR, *S. alba*, and *Brassica rapa*, all of which had damaged cotyledons. All olfactometer experiments were carried out between February and June 2023 in a climate‐controlled room (22 ± 0.6 °C, 60 ± 4% RH). Dual choice tests were completed with a glass Y‐tube olfactometer.

Preliminary tests using various types of olfactometers, including T‐tube olfactometer,^24^ still‐air olfactometer,^25^ four‐arm olfactometer,^26^ and six‐arm olfactometer^27^ and two sizes of Y‐tube olfactometers, were conducted. However, these setups consistently failed to achieve the desired outcomes, as the response rates of the beetles remained low across all trials.

The Y‐tube olfactometer (35 mm inner diameter) used here, consisted of a central arm (200 mm length) and two side arms (180 mm length) forming a 90° angle. A silicon plug (35 mm diameter, Rotilabo; Carl Roth GmbH, Karlsruhe, Germany) with a centrally positioned hole (7 mm diameter) was inserted into the open ends of each arm. The olfactometer was positioned inside a photo light box (60 cm × 60 cm × 60 cm; Yorbay eBusiness GmbH, Hamburg, Germany) on a green plastic platform inclined at an angle of 8°. A light source (LED, 5500K) was placed on the back wall of the box to provide an additional stimulus for beetle movement towards the side arms. Air supplied by an in‐house compressor was purified and humidified by passing through bottles of activated charcoal and water before reaching the flowmeters. The flowmeters regulated the airflow to 0.5 L min^−1^ before reaching the olfactometer arms. Tubing (6.4 mm ø, Tygon S3 E‐3603; Saint‐Gobain Ceramics & Plastics, Stow, OH, USA) was used to deliver the air stream between each part of the olfactometer system. The tubing and the silicon plugs in the olfactometer arms were connected by a glass cylinder (5 mm inner diameter).

Odor sources included 30 seedlings per pot or two, 6‐weeks old plants (BBCH 14) in one pot. Plants and pots were enclosed in a tube of 31 cm × 40 cm PET foil (Toppits Bratschlauch; Cofresco Frischhalteprodukte GmbH & Co. KG, Minden, Germany), with the two open ends of the foil tube taped to the tubing to ensure an airtight connection. The same method was applied for the control, which consisted of pots filled with the same substrate on the same day as the potted plants. To simulate CSFB feeding, plant material was positioned over a Petri dish lid for support and a Pasteur pipette was used to punch out holes (1 mm diameter). Two holes were made per cotyledon for seedlings (BBCH 10) and a total of 120 holes were made for larger plants (BBCH 14). This was done approximately 10 min before the start of each assay to induce the emission of glucosinolate degradation products. Mechanical damage was used instead of beetle feeding, because of a better standardization of damage area and timing. For testing individual compounds, the test compound was diluted at a ratio of 10^−3^ in silicon oil (M500; Carl Roth GmbH) and 20 μL of the diluted compound was applied to filter paper (2 cm × 3 cm; Whatman 3MM, Maidstone, UK). The treated filter paper was rolled and inserted into the air tube of the respective olfactometer arm, to deliver the volatiles with the air stream. Mixtures contained both compounds in equal amounts. Pure silicon oil on filter paper was used as a control.

A single beetle was released at the opening of the central arm, and the choice of the beetle was recorded when it first crossed the ‘finish line’ 10 cm from the end of one of the arms of the olfactometer. Beetles failing to cross a ‘finish line’ within 4 min after release were categorized as ‘non‐responding’ and excluded from statistical analysis. Each beetle was tested only once. To minimize positional bias, the position of the stimulus (left/right) was switched after testing three beetles. Clean olfactometers were used after every 20 beetles, plants were replaced after every ten beetles, and new filter papers were used every five beetles. Only beetles of one sex were tested in the same olfactometer to avoid any bias. The Y‐tubes were cleaned by rinsing with acetone and heated to 180 °C for 2 h. Six olfactometer experiments were completed in succession, using 40–60 beetles per sex and stimulus. The stimuli of an experiment were tested on the same days in randomized orders. Each experiment was conducted on at least three different days, between 09:00 and 16:00 to avoid day‐dependent effects.

### 2.4 Volatile collection and analysis

**Figure 1:**
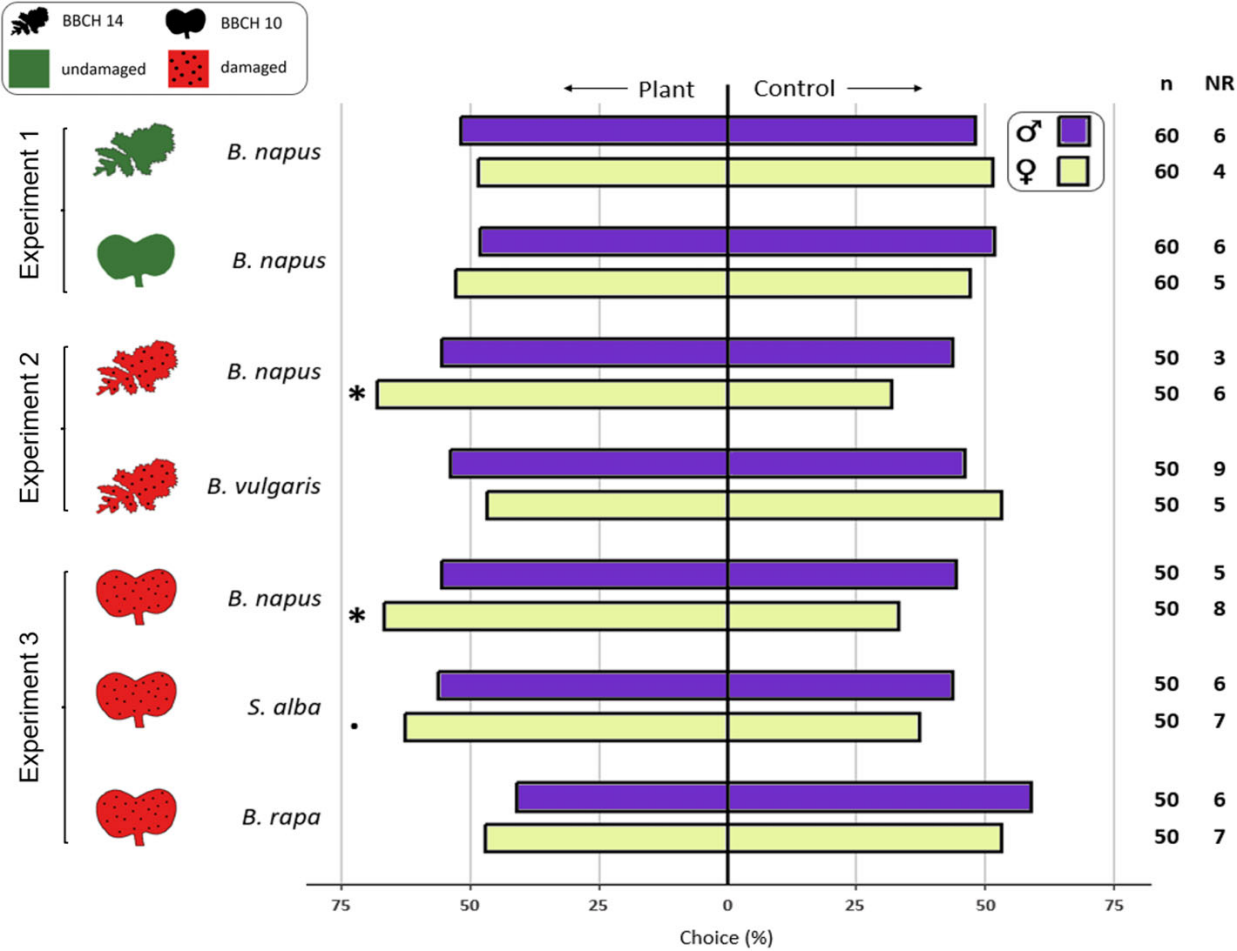
Olfactometer assay bioresults. Choice (%) of single adult *Psylliodes chrysocephala* (age: 3–6 days after emergence) in a Y‐tube olfactometer (8° inclined, airflow = 0.5 L min^−1^). Plants = plants potted in substrate; Test species: *Brassica napus, Beta vulgaris, Sinapis alba* and *Brassica rapa*. Damage was done mechanically by punching holes into the leaves. Control = pot with substrate. The sample size (*n*) and number beetles not reacting (NR) are given. A G‐test (expected ratio of 0.5:0.5) was used to analyze differences in responses. Significant differences are denoted with an asterisk (*P* < 0.05).

The volatile profiles emitted by the test plants were investigated by conducting headspace analyses using solid‐phase microextraction (SPME). Since undamaged plants did not elicit any chemotactic response in CSFB (Fig. 1), and Shannon *et al*.^28^ did not detect any VOCs emitted from undamaged OSR seedlings at BBCH 10, only damaged plants were used for VOC analysis. The sampling process was performed at a temperature of 20 ± 0.3 °C using a polydimethylsiloxane/divinylbenzene‐coated SPME‐fiber (65 μm, 57 310‐U; Supelco Inc., Bellefont, PA, USA). Prior to sampling, the fiber was desorbed at 220 °C for 5 min in the gas chromatograph inlet. To carry out the analysis, the plant material was placed inside a sealed glass vial (50 mL) with the opening covered by aluminum foil. For the analysis of volatiles from seedlings, 20 seedlings were damaged as described earlier. Seedlings were then cut at the base of the hypocotyl and placed inside the vial. For older plants, we removed a 3 cm × 3 cm section from each of the first three leaves and punctured 30 holes in each section using a Pasteur pipette as previously described, before placing them into the vial. A small hole was punctured in the aluminum foil, allowing the SPME fiber to be carefully inserted through it. After an adsorption period of 60 min, the fiber was withdrawn and immediately desorbed for analysis using gas chromatography–mass spectrometry (GC–MS, GC 7890B, MS 5977B; Agilent Technologies, Santa Clara, CA, USA), operating in splitless mode. The carrier gas for the GC was helium, with a flow rate of 1.5 mL min^−1^. A DB‐5 column was used, with a length of 30 m, an inner diameter of 0.25 mm, and a film thickness of 0.25 μm. The oven temperature was initially set at 40 °C for 3 min, then gradually increased to 220 °C and maintained at that temperature for 10 min. For tentative VOC identification, the MSD ChemStation software was used in conjunction with the Wiley 11 and NIST 17 mass spectral libraries. Retention indices and mass spectra were further verified by comparison with literature values and, where available, commercial standards.

### 2.5 Field experiment

A field experiment was completed at the end of June 2023 in an OSR field (cv. Bender, Deutsche Saatveredelung AG) near Göttingen, Germany (coordinates: 51°33'56.4"N, 9°56'45.8"E). The experiment used three individual volatile compounds in baited water traps to target CSFB shortly after their emergence from pupation. The OSR crop had been treated once with an insecticide (75 mL ha^−1^ Karate Zeon; Syngenta Agro GmbH, Frankfurt, Germany) at the end of March against pollen beetle (*Brassicogethes aeneus*). The experiment was set up in a randomized complete block design, with six blocks, 10 m between each baited trap within a block and at least 20 m between blocks. The traps were placed on the ground of the tractor tramlines. Each trap consisted of a yellow plastic tray (34 cm × 17 cm; Syngenta Agro GmbH) filled with water and a drop of odorless detergent (Pricol Ultra; Ecolab GmbH, Vienna, Austria) to reduce the water's surface tension. A volatile dispenser was attached to a wooden stick 10 cm above the center of the tray. The dispenser held a dental cotton roll (12 mm ø, 38 mm length) saturated with 0.8 g of the respective test compound (allyl‐ITC, 2‐phenylethyl ITC, or sec‐butyl ITC) and heat sealed into a polyethylene sachet (50 μm in thickness). In control traps, the cotton roll was left untreated. Captured CSFB were counted after 7 days. The daily mean temperature over the experimental course was 18.0 ± 0.8 °C with total precipitation of 1.2 mm occurring only on the first day.

### 2.6 Statistical analyses

Data analyses and visualizations were performed using R version 4.2.1 integrated into RStudio 2021.09.0 Build 351 (R core team 2021). The null hypothesis for the olfactometer experiments was an expected ratio of 0.5:0.5, tested with a G‐test of goodness‐of‐fit (package ‘AMR’). The field experiment was analyzed using a generalized linear model (family: Poisson) and a pairwise comparison of estimated marginal means (package ‘emmeans’) was used as *post hoc* test. All graphs were created with the ‘ggplot2’ package.

## 3 RESULTS

### 3.1 Olfactometer assays

**Figure 2:**
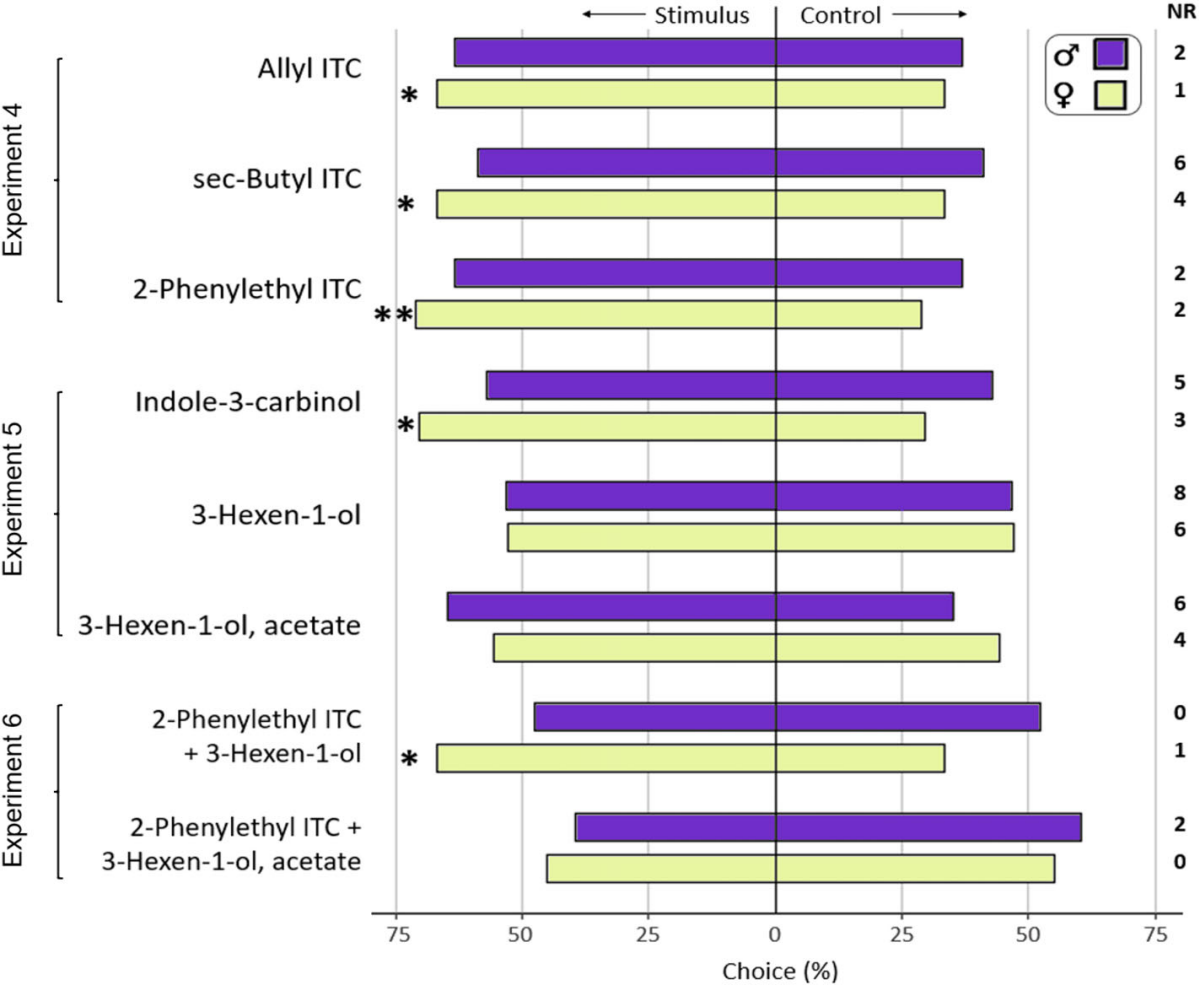
Results of olfactometer bioassays. Choice (%) of single adult *Psylliodes chrysocephala* (3–6 days after emergence) in a Y‐tube olfactometer (8° inclined, airflow = 0.5 L min^−1^). Stimulus = compound diluted 10^−3^ in silicon oil and 20 μL applied on filter paper. Control = 20 μL silicon oil on filter paper. The sample size per stimulus and sex was *n* = 40, number of beetles not responding (NR) is given. A G‐test (expected ratio of 0.5:0.5) was used to analyze for differences in responses. Significant differences (*P* < 0.05) are denoted with asterisks (*P < 0.05; **P < 0.01).

There was no significant difference in the response of adult CSFB to the odors of undamaged OSR plants or the control at either the cotyledon stage (females: *χ* ^*2*^ = 0.16, *P* = 0.686, DF = 1; males: *χ* ^*2*^ = 0.07, *P* = 0.786, DF = 1) or the four‐leaf stage (females: *χ* ^*2*^ = 0.07, *P* = 0.789, DF = 1; males: *χ* ^*2*^ = 0.07, *P* = 0.786, DF = 1), indicating no attraction (Experiment 1, Fig. 1). Only female CSFB were found to be significantly attracted to the odors from mechanically damaged OSR in the olfactometer (BBCH 10: *χ* ^*2*^ = 4.76, *P* = 0.029, DF = 1; BBCH 14: *χ* ^*2*^ = 5.95, *P* = 0.015, DF = 1) (Fig. 1). A high proportion (63%) of female beetles also oriented towards damaged *S. alba* seedlings, but this response was not significantly different from the control (*χ* ^*2*^ = 2.85, *P* = 0.092, DF = 1). Beetles were not attracted (females: *χ* ^*2*^ = 0.21, *P* = 0.647, DF = 1; males: *χ* ^*2*^ = 1.46, *P* = 0.227, DF = 1) to *Brassica rapa* seedlings (Fig. 1). When tested with pure volatile compounds in the olfactometer, females were significantly attracted to allyl ITC (*χ* ^*2*^ = 4.42, *P* = 0.036, DF = 1), sec‐butyl ITC (*χ* ^*2*^ = 4.08, *P* = 0.043, DF = 1), 2‐phenylethyl ITC (*χ* ^*2*^ = 6.95, *P* = 0.008, DF = 1) and indole‐3‐carbinol (*χ* ^*2*^ = 6.26, *P* = 0.012, DF = 1) (Fig. 2). With 71% of the females orienting towards the 2‐phenylethyl ITC odor source, this was the strongest response observed in the olfactometer. When 2‐phenylethyl ITC was offered together with 3‐hexen‐1‐ol, females also showed a positive response to this stimulus over the control (*χ* ^*2*^ = 4.42, *P* = 0.036, DF = 1), but this was not the case when 3‐hexen‐1‐ol was tested alone (*χ* ^*2*^ = 0.12, *P* = 0.732, DF = 1) or when 2‐phenylethyl ITC was mixed with 3‐hexen‐1‐ol, acetate (*χ* ^*2*^ = 0.4, *P* = 0.505, DF = 1) (Fig. 2). Male CSFB showed no significant orientation to any of the tested odors in the olfactometer experiments (Figs 1 and 2).

### 3.2 Volatile analysis

The volatile blends from mechanically damaged OSR (BBCH 10 and BBCH 14), *Beta vulgaris* (BBCH 14), *S. alba* (BBCH 10) and *Brassica rapa* (BBCH 10) were analyzed. A total of 43 volatile compounds were detected, with 30 compounds putatively identified (Table 1). The green leaf volatiles 1‐penten‐3‐ol, 3‐hexen‐1‐ol and 3‐hexen‐1‐ol acetate were consistently detected in all samples. *Brassica rapa* exhibited the highest diversity in VOCs, emitting 23 different compounds, whereas *S. alba* seedlings showed the lowest VOC diversity, with only seven compounds detected (Table 1). The predominant compound emitted by both stages of OSR was 3‐hexen‐1‐ol acetate, while 3‐hexen‐1‐ol emerged as the most abundant compound in all other species. ITCs could only be identified in the headspace of *Brassica rapa* seedlings. *Brassica rapa* was found to emit six nitriles/epithionitriles, whereas OSR (BBCH 14) emitted two, and *S. alba* and *Beta vulgaris* only one compound of this class.

**Table 1:** Volatile organic compounds identified from the headspace of mechanically damaged plants (BBCH 14) and seedlings (BBCH 10) of *Brassica napus*, *Beta vulgaris*, *Sinapis alba* and *Brassica rapa*

Compound	Rt	RI	*Brassica napus* (BBCH 14)	*Beta vulgaris* (BBCH 14)	*Brassica napus* (BBCH 10)	*Sinapis alba* (BBCH 10)	*Brassica rapa* (BBCH 10)
*Alcohols*							
1‐Penten‐3‐ol^†^	3.57	662	5.15 ± 3.67	4.2 ± 1.24	0.71 ± 0.1	1.45 ± 0.52	2.57 ± 1.31
3‐Hexen‐1‐ol^†^	7.04	850	255.05 ± 57.1	424.81 ± 71.36	11.33 ± 4.79	15.86 ± 6.0	228.65 ± 15.48
2‐Hexen‐1‐ol (*E*)	7.26	861	—	24.95 ± 6.32	—	—	—
3‐Methyl‐4‐penten‐1‐ol	7.30	863	—	—	—	—	9.52 ± 0.51
*Aldehydes*							
2‐Hexenal	5.75	802	—	6.68 ± 3.60	—	2.61 ± 1.06	—
Decanal	14.21	1210	—	0.46 ± 0.12	—	—	1.43 ± 0.46
*Esters*							
2‐Penten‐1‐ol acetate	8.27	909	12.41 ± 3.77	—	—	—	14.43 ± 3.02
3‐Hexen‐1‐ol acetate^†^	10.28	1005	488.87 ± 61.42	274.94 ± 36.13	28.61 ± 10.21	4.43 ± 1.97	350.14 ± 35.52
Hexyl acetate	10.43	1012	—	18.78 ± 2.49	—	—	30.11 ± 7.17
*cis*‐3‐Hexenyl isobutyrate	13.00	1145	11.82 ± 8.34	—	—	—	—
*cis*‐3‐Hexenyl butyrate	13.81	1188	21.62 ± 16.46	—	—	—	—
*cis*‐3‐Hexenyl isovalerate^†^	14.66	1236	1.75 ± 0.64	—	—	—	0.52 ± 0.08
Hex‐3‐enyl propanoate	12.18	1101	28.46 ± 19.58	—	—	—	3.19 ± 0.97
*Isothiocyanates*							
*sec*‐Butyl isothiocyanate^†^	8.74	931	—	—	—	—	17.48 ± 7.02
3‐Butenyl isothiocyanate	9.86	985	—	—	—	—	20.99 ± 7.91
5‐Isothiocyano‐1‐pentene	11.91	1087	—	—	—	—	60.62 ± 20.58
1‐Isothiocyanato‐heptane	15.27	1271	—	—	—	—	2.00 ± 0.42
*Nitriles and epithionitriles*							
2‐Methylbutanenitrile^†^	4.21	723	3.41 ± 1.55	—	—	—	48.01 ± 7.54
3‐Methyl‐2‐butenenitrile	5.07	767	—	—	—	—	1.64 ± 0.38
5‐Cyano‐1‐pentene^†^	7.16	856	—	—	—	—	21.78 ± 4.34
4‐Cyanocyclohexene^†^	10.65	1024	5.65 ± 1.17	3.29 ± 0.69	4.83 ± 2.63	2.22 ± 0.92	—
Azeleonitrile	11.13	1048	‐	‐	‐	‐	3.28 ± 0.64
Cyano‐3,4‐epithiobutane	12.75	1131	—	—	—	—	26.01 ± 16.67
1‐Cyano‐4,5‐epithiopentane	15.04	1258	—	—	—	—	21.98 ± 12.44
*Others*							
3‐Ethyl‐1,5‐octadiene	8.97	942	—	—	—	—	1.13 ± 0.14%
Dodecane^†^	14.04	1201	—	—	0.24 ± 0.08	—	0.81 ± 0.26
6‐Methyl‐5‐hepten‐2‐one	9.9	987	—	2.88 ± 0.62	—	—	—
Eucalyptol	10.86	1034	3.39 ± 1.26	—	—	—	—
2,4‐Hexadien‐1‐ol	4.33	729	—	0.57 ± 0.09	—	—	—
Cyclic octaatomic sulfur	26.53	2087	—	—	—	0.11 ± 0.03	—
*Unidentified*							
Unknown 1	8.08	900	4.63 ± 0.17	1.47 ± 0.29	1.39 ± 0.35	1.17 ± 0.25	—
Unknown 2	8.34	912	—	1.3 ± 0.43	—	—	—
Unknown 3	8.38	914	2.58 ± 0.36	—	—	—	—
Unknown 4	9.3	958	0.38 ± 0.12	—	0.34 ± 0.09	—	—
Unknown 5	10.49	1015	—	7.14 ± 0.52	—	—	—
Unknown 6	10.77	1030	4.34 ± 2.49	—	—	—	3.07 ± 0.62
Unknown 7	12.29	1106	—	0.72 ± 0.06	—	—	—
Unknown 8	12.51	1118	—	1.00 ± 0.08	—	—	—
Unknown 9	16.24	1328	—	—	—	—	2.21 ± 0.9
Unknown 10	17.43	1401	0.39 ± 0.19	0.11 ± 0.02	—	—	—
Unknown 11	18.36	1461	—	0.29 ± 0.09	—	—	—
Unknown 12	20.19	1584	—	0.18 ± 0.04	—	—	—
Unknown 13	25.42	1985	—	—	0.13 ± 0.05	—	—

*Note*: Rt, retention time; RI, retention index; Compounds were quantified as relative peak area (mean ± standard error, divided by 10 000 000).

^†^ Identified by comparison with authentic standard.

### 3.3 Field experiment

**Figure 3:**
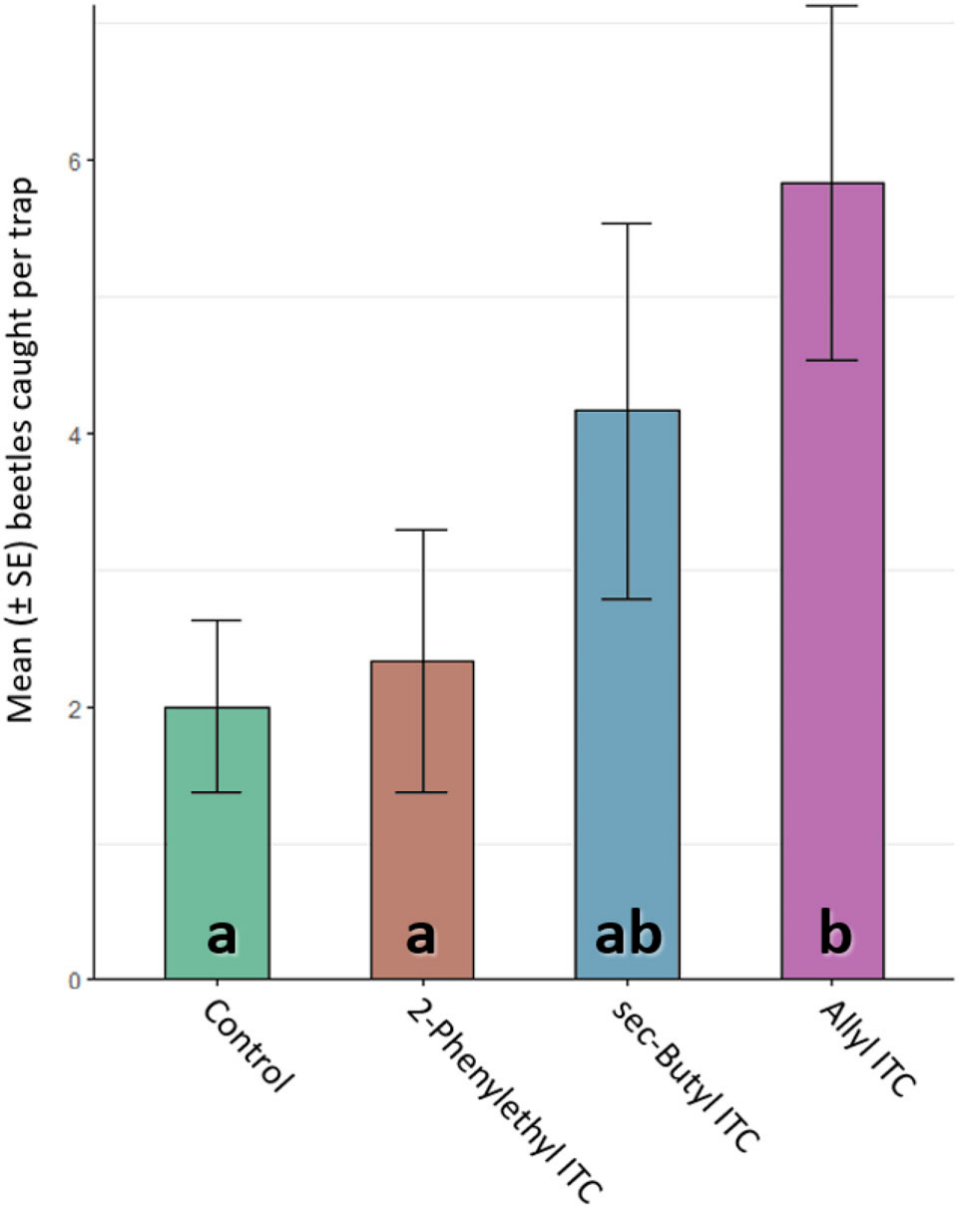
Results of the field experiment. Mean [± standard error] number of adult *Psylliodes chrysocephala* caught in baited traps placed in an oilseed rape field for 7 days in June 2023. For baiting, a sachet dispenser (cotton roll impregnated with 0.8 g of test compound, heat sealed in a polyethylene bag of 50 μm thickness) was hung over a yellow water trap. Different letters indicate significant differences (*α* = 0.05) by a generalized linear model (family: Poisson) and pairwise comparison of estimated marginal means.

In the field experiment, only traps baited with allyl ITC caught significantly more beetles than the control (*P* = 0.008) (Fig. 3). Allyl ITC also caught significantly more beetles than 2‐phenylethyl ITC (*P* = 0.02). On average, twice as many beetles were caught in traps baited with sec‐butyl ITC, but this was not significantly different from the control due to large variation in numbers of beetles trapped (*P* = 0.156) (Fig. 3).

## 4 DISCUSSION

The CSFB has been the subject of research for decades owing to its significant impact on OSR cultivation. Despite its destructive potential, there is a paucity of published data on the olfactory behavior of this pest, possibly because researchers had difficulty observing such behavior in the laboratory. Our research aimed to address this gap by investigating the attraction of adult CSFB to plant odors in both controlled experiments in a laboratory setting and in field conditions. Using a Y‐tube olfactometer, we showed that only mechanically damaged OSR seedlings and plants with four true leaves elicited significant attraction, and only when female beetles were used; behavioral responses of males were not significantly different between host/non‐host plant stimuli and controls. Additionally, we tested volatile compounds in the field, providing a practical means of evaluating CSFB olfactory responses in real‐world agricultural environments.

When exposed to plant stimuli, attraction was observed only if OSR plants and seedlings had been subjected to mechanical damage. We cannot rule out the possibility that beetles could not detect the low volume of plant material used in seedling experiments, however, the absence of attraction towards undamaged OSR plants may underline the importance of ITCs as host plant cues, since ITCs are only formed upon cell damage.^14^ However, headspace analyses failed to detect any ITCs from damaged OSR plants. Shannon *et al*.^28^ similarly did not detect glucosinolate‐derived volatiles from OSR seedlings at growth stages BBCH 10 and BBCH 11–12 when using undamaged seedlings. Nevertheless, the emission of ITCs is likely, and detection probably failed due to low emission quantities, since only small amounts of glucosinolates which may hydrolyze to ITCs were found in cotyledons of cv. Arabella (Rüde D, unpublished data). In contrast, cotyledons of *S. alba* were found to contain a high amount of the glucosinolate sinalbin (Rüde D, unpublished data). Sinalbin undergoes enzymatic conversion to 4‐hydroxybenzyl ITC, which, due to its instability, may further react to form non‐volatile 4‐hydroxybenzyl alcohol and thiocyanate.^29^ This chemical cascade could potentially explain the absence of 4‐hydroxybenzyl ITC in our samples of mechanically damaged *S. alba* cotyledons.

Isothiocyanates are known to serve as host plant cues for several OSR pests of different orders.^12^, ^30^, ^31^, ^32^ Field experiments have shown that CSFB are attracted to allyl ITC^33^ and a mixture of ITCs.^17^ To extend the knowledge on the role of ITCs for CSFB host location, we tested three ITCs and indole‐3‐carbinol, which results from the unstable indole‐3‐methylisothiocyanate and is the related ITC of glucobrassicin.^34^ Allyl ITC was chosen due to its low cost and easy availability, making it promising as a lure for monitoring traps. Sec‐butyl ITC was tested as we found it in the headspace analysis of *Brassica rapa* seedlings. 2‐Phenylethyl ITC is the related ITC of the glucosinolate gluconasturtiin, which can be found in cotyledons^35^ and true leaves of OSR,^36^ and so it was likely to be emitted by the damaged OSR tested. 3‐Hexen‐1‐ol and 3‐hexen‐1ol acetate were present in the headspaces of all tested plants and both are known to be common green leaf volatiles.^37^

We showed that indole‐3‐carbinol and all tested ITCs were attractive to CSFB in olfactometer tests and so we were interested in how responses differed to mixtures with green leaf volatiles. We found a mixture of 2‐phenylethyl ITC and 3‐hexen‐1‐ol to be attractive, but interestingly, no effect was found when 2‐phenylethyl ITC was presented simultaneously with 3‐hexen‐1‐ol acetate. Although 2‐phenylethyl ITC was attractive in the olfactometer, it failed to elicit attraction of adult CSFB to traps in the field. This might be due to a competitive effect of 2‐phenylethyl ITC, which probably was emitted by the surrounding OSR plants and thus reducing orientation towards the trap. That 2‐phenylethyl ITC was emitted by the surrounding OSR plants is very likely, since the related glucosinolate has been shown to be present in that stage of OSR.^36^ Baiting with allyl ITC increased the number of CSFB in traps, which was also found by Tóth and Csonka.^33^

Attraction to host plant VOCs in laboratory experiments was only found for female CSFBs. Bartlet *et al*.^38^ did not find a sexual dimorphism in the number, distribution, or structure of the CSFB antennal sensilla, but sexual differences in the behavioral response towards volatile cues is a known phenomenon. In a meta‐analysis of the effect of plant volatiles on insect pests, Szendrei and Rodriguez‐Saona^39^ found that males were significantly less attracted to traps baited with plant volatiles than females. Groot *et al*.^40^ found sexual differences in the electrophysiological response of *Lygocoris pabulinus* towards different volatile classes, concluding a higher sensitivity of female *Lygocoris pabulinus* to plant VOCs due to their orientation towards oviposition sites, while male sensitivity is higher to pheromone‐type compounds.

No attraction towards damaged *Brassica rapa* seedlings was observed, contrary to expectations considering the emission of several glucosinolate‐derived volatiles, which could have served as host–plant cues for CSFB. Moreover, Barari *et al*.^20^ reported that *Brassica rapa* was highly attractive to female CSFB for oviposition, demonstrating that *Brassica rapa* plants were more attractive than *Brassica napus*. This divergence in the findings between Barari *et al*.^20^ and our study could potentially be attributed to the distinct volatile compositions at different growth stages, highlighting the need for a nuanced understanding of plant attractiveness dynamics across various developmental phases.^6^ The absence of attraction to *Brassica rapa* odors in our experiments may have been caused by various factors. It is possible that specific VOCs neutralized the attraction mediated by glucosinolate‐derived volatiles, or that the host–plant cues emitted by *Brassica rapa* were overly abundant, resulting in an inhibition of attraction.^41^

In our olfactometer experiments, the beetle's developmental stage was pre‐aestivation, that is, they were tested a few days after emergence from pupation. Aestivation is a summer diapause, during which the energy metabolism of CSFB changes drastically and heat tolerance increases.^42^ In the field, immigration of CSFB into new OSR crops occurs after aestivation.^3^ The developmental stage of an insect might alter the detection or the elicited behavior of certain VOCs, as found for the related flea beetle *Phyllotreta cruciferae*.^24^, ^43^ Background and habitat cues, in addition to VOC blends and the ratios of their components can influence the host finding of insects by volatile cues.^44^, ^45^ Changes in the ratio of VOC blends can significantly influence insect host location, as demonstrated by Visser and Avé.^46^ Their study on *Leptinotarsa decemlineata* revealed that even small variations in the ratios of a green leaf volatile blend disrupted attraction when the ratios were unnatural. Attraction towards certain compounds may also be dose‐dependent.^47^, ^48^ These factors should be investigated in future studies, to better understand the host finding process of CSFB.

In this study, we investigated the behavioral response of adult CSFB to volatiles emitted by brassicaceous plants and a non‐host. Our findings indicate that female CSFB exhibit orientation towards host plant odors, particularly to damaged plants of *Brassica napus*. Glucosinolate‐derived volatiles, such as 2‐phenylethyl ITC, indole‐3‐carbinol, allyl ITC, and sec‐butyl ITC significantly attracted female CSFB in olfactometer tests. Baiting conventional field traps with allyl ITC increased the number of captured CSFB compared to unbaited control traps. To our knowledge, these experiments are the first to show behavioral responses of adult CSFB towards volatiles of brassicaceous plants in olfactometer tests. The Y‐tube olfactometer setup described here can be utilized in future investigations to efficiently screen plants for reduced attractiveness, which is valuable for enhancing plant breeding.^4^ Additionally, it can be used to identify compounds needed as effective lures for integrated pest management strategies.^1^ However, the question of how CSFB locate OSR fields after aestivation remains unanswered.

## CONFLICT OF INTEREST STATEMENT

The authors have no conflicts of interest.

## Data Availability

The data that support the findings of this study are available from the corresponding author upon reasonable request.
